# De novo assembly and analysis of the transcriptome of the Siberian wood frog Rana amurensis

**DOI:** 10.18699/VJGB-22-07

**Published:** 2022-02

**Authors:** D.N. Smirnov, S.V. Shekhovtsov, A.A. Shipova, G.R. Gazizova, E.I. Shagimardanova, N.A. Bulakhova, E.N. Meshcheryakova, T.V. Poluboyarova, E.E. Khrameeva, S.E. Peltek, D.I. Berman

**Affiliations:** Center of Life Sciences, Skolkovo Institute of Science and Technology, Moscow, Russia; Ben-Gurion University of the Negev, Department of Life Sciences, Beer Sheva, Israel; Institute of the Biological Problems of the North of the Far-Eastern Branch of the Russian Academy of Sciences, Magadan, Russia; Institute of Cytology and Genetics of the Siberian Branch of the Russian Academy of Sciences, Novosibirsk, Russia; Institute of Cytology and Genetics of the Siberian Branch of the Russian Academy of Sciences, Novosibirsk, Russia; Institute of Fundamental Medicine and Biology, Kazan Federal University, Kazan, Russia; Institute of Fundamental Medicine and Biology, Kazan Federal University, Kazan, Russia; Institute of the Biological Problems of the North of the Far-Eastern Branch of the Russian Academy of Sciences, Magadan, Russia; Institute of the Biological Problems of the North of the Far-Eastern Branch of the Russian Academy of Sciences, Magadan, Russia; Institute of Cytology and Genetics of the Siberian Branch of the Russian Academy of Sciences, Novosibirsk, Russia; Center of Life Sciences, Skolkovo Institute of Science and Technology, Moscow, Russia; Institute of Cytology and Genetics of the Siberian Branch of the Russian Academy of Sciences, Novosibirsk, Russia; Institute of the Biological Problems of the North of the Far-Eastern Branch of the Russian Academy of Sciences, Magadan, Russia

**Keywords:** Siberian wood frog, Rana amurensis, transcriptome, de novo assembly, neurotransmitters, сибирская лягушка, Rana amurensis, транскриптом, сборка de novo, нейромедиаторы

## Abstract

The Siberian wood frog Rana amurensis Boulenger, 1886 is the most hypoxia-tolerant amphibian. It can survive for several months in an almost complete absence of oxygen. Little is known about the mechanisms of this remarkable resilience, in part because studies of amphibian genomes are impeded by their large size. To make the Siberian wood frog more amenable for genetic analysis, we performed transcriptome sequencing and de novo assembly for the R. amurensis brain under hypoxia and normoxia, as well as for the normoxic heart. In order to build a de novo transcriptome assembly of R. amurensis, we utilized 125-bp paired-end reads obtained from the brain under normoxia and hypoxia conditions, and from the heart under normoxia. In the transcriptome assembled from about 100,000,000 reads, 81.5 % of transcripts were annotated as complete, 5.3 % as fragmented, and 13.2 % as missing. We detected 59,078 known transcripts that clustered into 22,251 genes; 11,482 of them were assigned to specific GO categories. Among them, we found 6696 genes involved in protein binding, 3531 genes involved in catalytic activity, and 576 genes associated with transporter activity. A search for genes encoding receptors of the most important neurotransmitters, which may participate in the response to hypoxia, resulted in a set of expressed receptors of dopamine, serotonin, GABA, glutamate, acetylcholine, and norepinephrine. Unexpectedly, no transcripts for histamine receptors were found. The data obtained in this study create a valuable resource for studying the mechanisms of hypoxia tolerance in the Siberian wood frog, as well as for amphibian studies in general.

## Abstract (ru)

Сибирская лягушка Rana amurensis Boulenger, 1886 – наиболее устойчивый к гипоксии вид амфибий. Она может прожить несколько месяцев при почти полном отсутствии кислорода. О механизмах этой замечательной устойчивости мало что известно, отчасти потому, что исследования геномов амфибий затруднены из-за их большого размера. Чтобы сделать сибирскую лягушку более доступной для генетического анализа, мы провели секвенирование и сборку de novo транскриптома мозга R. amurensis в условиях гипоксии и нормоксии, а также для сердца – в нормоксии. Для сборки транскриптома de novo использовали парные прочтения длиной 125 п. н., полученные для мозга сибирской лягушки в нормоксии и гипоксии, а также для сердца контрольных особей. В транскриптоме, собранном из примерно 100 млн ридов, 81.5 % транскриптов были аннотированы как полные, 5.3 – как фрагментированные и 13.2 % – как отсутствующие. Мы обнаружили 59 078 известных транскриптов, которые были сгруппированы в 22 251 ген, 11 482 из них были отнесены к определенным категориям Gene Ontology. Среди них – 6696 генов, участвующих в белок-белковом взаимодействии, 3531 ген, кодирующий белок с каталитической функцией, и 576 генов, связанных с транспортной активностью. Для большинства транс- криптов были установлены тканеспецифичные различия в экспрессии. Известно, что нейротрансмиттеры игра- ют важную роль в ответе на гипоксию различных организмов, устойчивых к недостатку кислорода. Поиск генов, кодирующих рецепторы важнейших нейромедиаторов, которые могут участвовать в реакции на гипоксию, выявил набор экспрессируемых рецепторов дофамина, серотонина, гамма-аминомасляной кислоты (ГАМК), глутамата, ацетилхолина и норадреналина. При этом не обнаружено транскриптов для рецепторов гистамина. Данные, полученные в нашей работе, представляют собой ценный ресурс для исследования механизмов толе- рантности к гипоксии у сибирской лягушки, а также для изучения амфибий в целом.

## Introduction

Next-generation sequencing revolutionized the studies in the field of molecular genetics. In contrast to early whole-genome projects, this technology presents a quick and relatively cheap way to obtain genome-wide information for non-model organisms. However, for organisms with large genome sizes, such as amphibians, this is still a challenge due to many repeat sequences, frequent cases of polyploidy and high costs associated with the sequences of large genomes (Schatz et al., 2010). Among the family Ranidae, there are currently only three genome assemblies: Rana temporaria, Glandirana rugosa, and Lithobates catesbeianus (Hammond et al., 2017; Katsura et al., 2021; Streicher et al., 2021). Available transcriptomes are more numerous; however, they are still provided only for a limited number of members of the family Ranidae and do not always meet the high-quality standards of modern transcriptome assemblies.

Assembled transcriptomes would be useful resources for studies on the emergent model species. Among these species are the northern amphibians that adapted to extreme conditions of the northern Palearctic. These include highly freeze-tolerant Rana sylvatica LeConte, 1825 (Storey, 1984), R. arvalis Nilsson, 1842 (Berman et al., 2020), Hyla japonica Günther, 1859 (Berman et al., 2016a), and the urodelas Salamandrella keyserlingii Dybowski, 1870 and S. schrenkii (Strauch, 1870) (Berman et al., 1984, 2010, 2016b), as well as the hypoxiatolerant Siberian wood frog Rana amurensis Boulenger, 1886 (Berman et al., 2019). These species are intensely studied because they represent one of the most remarkable adaptations of vertebrates to extreme conditions and could give insights into ischemia treatment and organ transplantation. Earlier studies of freeze- and hypoxia tolerant amphibians were mostly aimed at their physiology and biochemistry, but studying genetic systems becomes more important (Bickler, Buck, 2007; Storey K.B., Storey J.M., 2017).

Amphibians in general are believed to be not particularly tolerant to hypoxia: adults of the different studied species can survive for a few hours to a few days even at low (near-zero) temperatures in water with low oxygen content (Bickler, Buck, 2007). However, the Siberian wood frog R. amurensis Boulenger, 1886 is unique among amphibians in its ability to survive almost complete anoxia for several months (Berman et al., 2019). This makes it a promising model object for studying hypoxia tolerance. Metabolomic patterns in its organs indicate dramatic changes in biochemical pathways under hypoxia (Shekhovtsov et al., 2020). However, these patterns are not easy to interpret, and this could be facilitated by the analysis of gene expression and gene networks. In order to create a resource for studying gene expression in the Siberian wood frog, we performed sequencing, de novo assembly, and annotation of the transcriptome of this species.

Brain and heart are the most sensitive to hypoxia (Nilsson et al., 2015; Swenson, 2016), so we used transcripts from these organs for transcriptome construction. To test the assembled transcriptome, we also performed a search for neurotransmitter receptor genes: it was demonstrated (Nilsson et al., 1990, 1991) that neurotransmitters mediate hypoxia response in turtles, so we hypothesized that this might also be true for the Siberian wood frog.

## Materials and methods

RNA extraction and sequencing. Specimens of the Siberian wood frog were collected in September 2019 near the Lesopilnoye village, Khabarovsk Krai (46° N, 134° E). We followed approved methods under appropriate permits issued by cognizant governmental agencies (No. 001/04-19). Frog handling, hypoxia exposure, and organ extraction were performed as described in S.V. Shekhovtsov et al. (2020): briefly, the frogs were distributed by 5–7 individuals into 10 L containers filled with water (oxygen level 7–8 mg/L) and acclimated to low temperatures: 2 days at 14–15 °C, for 4 days at 8, 4, and 2–3 °C. Acclimation was performed in a TSO-1/80 SPU thermostat (SKTB SPU, Russia) and in a WT-64/75 climatic test chamber (Weiss Umwelttechnik GmbH, Germany). Control animals were kept in open containers; those exposed to hypoxia, in closed airtight bottles. The dissolved oxygen content was measured daily by a HACH HQ30D Flexi digital single-channel device with a luminescent LDO101 sensor until it reached 0.2 mg/L. After 17 days in hypoxia, animals were slaughtered as quickly as possible, and the organs were extracted and immediately submerged in liquid nitrogen. RNA was extracted using commercial kits (Biolabmix, Russia) following the manufacturer’s protocol

The purity of total RNA was estimated on a NanoPhotometer (Implen, Germany). The quantity of total RNA was measured by fluorimeter Qubit 4.0 (ThermoFisher Scientific, USA). The quality of total RNA was evaluated using a Bioanalyzer 2100 (Agilent Technologies, USA). Then, from 800–1000 ng of pure and good quality total RNA (RIN ≥ 7), polyA mRNA was isolated using NEBNext Poly(A) mRNA Magnetic Isolation Module (New England Biolabs, USA).

cDNA libraries were prepared using NEBNext Ultra II Directional RNA Library Prep Kit for Illumina (New England Biolabs) according to the manufacturer’s protocol. The concentration of amplified libraries was estimated by fluorimeter Qubit 3.0 (ThermoFisher Scientific). Size selection of pooled libraries was performed on the BluePippin system (SAGE Science, USA) using 1.5 % agarose gel cassettes with 300–400 bp target size. The quality of libraries was verified on a Bioanalyzer 2100 (Agilent Technologies) using DNA High Sensitivity Kit. Range of library fragment size was 200–1000 bp. The concentration of libraries was validated by qPCR using 2.5× EVA Green Mix (Synthol, Russia) and primers for Illumina adapters (Evrogen, Russia). Libraries were then sequenced on a HiSeq 2500 (Illumina, USA) with paired-end 125 bp reads

Transcriptome assembly. RNA reads from R. amurensis brain and heart samples (brain+normoxia, brain+hypoxia, heart+normoxia, 3 samples in total) were used for de novo transcriptome assembly. The quality of raw reads was estimated with FastQC (https://www.bioinformatics.babraham. ac.uk/projects/fastqc/). Adaptor trimming and read filtering were performed using fastp (Chen et al., 2018) with default parameters. The rCorrector tool (Song, Florea, 2015) was used for correcting the non-solid k-mers within reads and removing unfixable ones. Transcriptome assembly was performed on all samples via Trinity (Grabherr et al., 2011) with --SS_lib_type FR parameter for a stranded library. The basic assembly metrics were calculated using the ‘TrinityStats. pl’ script incorporated in Trinity. Redundant transcripts were identified and removed from the assembly via CD-HIT (Fu et al., 2012) with the following parameters: -c 0.98 -p 1 -d 0 -b 3 -T 5 -M 2000. Completeness of the assembled transcriptome was estimated using BUSCO (Simão et al., 2015) with the mode -m transcriptome and lineage “tetrapoda_odb10” parameters.

Transcript quantification. The transcript abundance was estimated using the ‘align_and_estimate_abundance.pl’ script (--est_method salmon) included in Trinity. Both gene- and isoform-level abundance matrices for all samples were constructed with the ‘abundance_estimates_to_matrix.pl’ script. The comparison of samples based on their expression level and the subsequent visualization procedures were performed using the ‘PtR’ script as well as custom scripts.

Assembly annotation and candidate coding regions identification. We used a collection of scripts from TransDecoder (Grabherr et al., 2011) to identify the candidate coding regions. First, open reading frames (ORF) were retrieved from the assembly file. A set of the longest obtained ORFs were then queried against Swiss-Prot (Bairoch, Apweiler, 1999; The Uniprot Consortium, 2021) and Pfam (Mistry et al., 2021) databases to search for sequence similarity with known proteins and Pfam protein domains. To achieve better computational efficiency, we used hmmsearch v3.3.2 (Eddy, 2011) scripts instead of hmmscan for domain identification, and the homology search was done with Blast+ (Camacho et al., 2009). The output generated from the database searching step was then used for the prediction of coding regions using the TransDecoder.Predict script from TransDecoder and for transcriptome assembly annotation via the Trinotate (Bryant et al., 2017) pipeline

Gene Ontology (GO) analysis. The Trinotate report obtained in the assembly annotation step was used to characterize the annotated genes according to their biological role and the occupied cell compartments. We counted the number of annotated genes per GO category for the cellular component (CC), biological process (BP), and molecular function (MF) sub-ontologies at level 2. Graphical representation was done using an in-house R script. Annotated genes without assigned GO categories were classified according to the PFAM protein families they associated with.

Searching for neurotransmitter receptors. We extracted a set of genes encoding receptors of the main neurotransmitters (dopamine, serotonin, GABA, glutamate, acetylcholine, histamine, and norepinephrine) from the Xenopus genome database (Xenbase; http://www.xenbase.org). Xenbase was chosen over more closely related species due to its longer history and better annotation. For each annotated gene, the transcripts were taken for Xenopus tropicalis, or, if absent, for X. laevis. We performed a blastn search for this Xenopus transcript dataset in the assembled transcriptome (Trinity_filtered.fasta) with e-value <1e–5. Transcripts with > 70 % sequence similarity were included in the final dataset.

## Results and discussion

De novo transcriptome assembly

In order to build a de novo transcriptome assembly of R. amurensis, we utilized 125 bp paired-end reads obtained from the brain under normoxia and hypoxia conditions (RABN and RABH samples, respectively) and from the heart under normoxia (RAHN sample).

After filtering out low-quality reads, a total of 98,948,825 reads from all three samples were used for the subsequent transcriptome assembling procedure. An initial assembly consisted of 610,890 Trinity ‘genes’ composed of 839,939 transcripts with an average contig length of 639 bp (or 481 bp based on the longest isoform per Trinity ’gene’). In addition, we filtered out 56,044 redundant transcripts using CD-HIT. Once filtering was done, the final assembly was generated (Table 1).

**Table 1.**
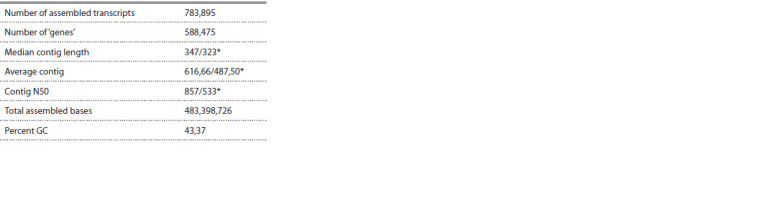
The statistics of the final Trinity assembly * Indicates values for statistics based on the longest isoform per Trinity ‘gene’.

Estimates using BUSCO demonstrated that 81.5 % of the transcripts were annotated as complete, 5.3 % as fragmented, and 13.2 % as missing.

Abundance quantification

**Fig. 1.**
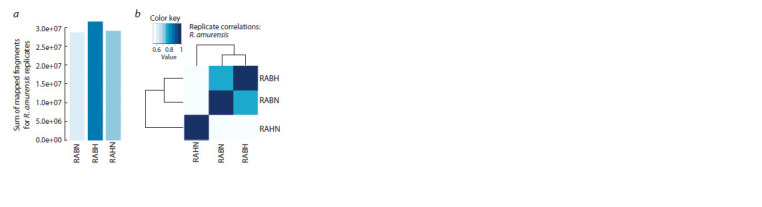
Comparative analysis of expression of R. amurensis samples. a – barplot representing the distribution of the total number of mapped fragments across all samples; b – heatmap showing hierarchical clustering of samples based on their expression levels. In both panels, RABH corresponds to R. amurensis brain sample under hypoxia; RABN – R. amurensis brain sample under normoxia, and RAHN is a R. amurensis heart sample under normoxia.

For each RNA sample, we calculated the levels of transcript abundances using the resulting assembled transcriptome. The Salmon alignment rate varied from 87.9 to 91 % across all samples, which is an additional indicator of good quality of the final assembly. The majority of Trinity ‘genes’ turned out to be low-expressed, and only 20,251 out of 588,475 ‘genes’ had expression levels ≥ 10 TPM (transcripts per million) in at least one sample. The sum of gene expression counts per sample varied from 28,587,718 to 31,477,299 with the largest value for the R. amurensis brain sample under hypoxia (Fig. 1, a).

Moreover, we observed tissue-specific differences in gene expression levels between brain and heart samples (see Fig. 1, b).

**Fig. 2.**
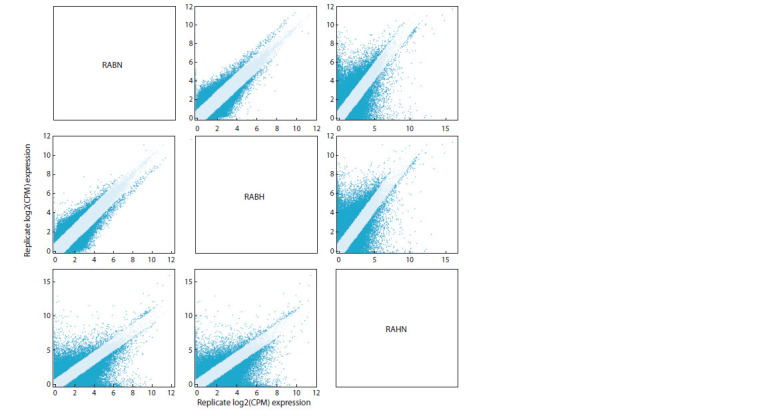
Sample-to-sample scatter plots showing gene expression differences between R. amurensis samples. Circles corresponding to 2-fold changes are marked in blue.

As was expected, we found that the brain transcriptomes correlated better with each other (Pearson’s r = 0.770) than with the heart transcriptome (Pearson’s r = 0.538 and 0.535 for RAHN correlation with RABN and RABH, respectively). In addition, we counted the number of genes with more than 2-fold expression change between each pair of samples (Fig. 2). For brain-brain transcriptome comparisons, the number of such genes was equal to 34,488, while for brain-heart comparisons, this number almost doubled (70,005 genes for RABN versus RAHN and 68,497 genes for RABH versus RAHN). Taken together, all these findings on gene and transcript quantification indicate the correctness of the transcriptome assembly.

Transcriptome assembly and annotation

**Fig. 3.**
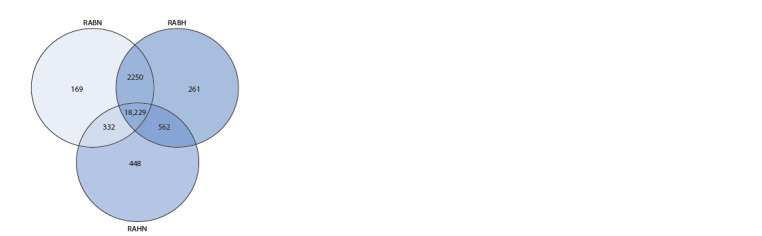
Venn diagram showing the number of common and samplespecific expressed genes between R. amurensis samples.

Once the expression quantification step was done, we annotated the obtained transcripts to evaluate the number of biologically relevant ones. We first identified a total of 141,950 candidate coding regions using TransDecoder and obtained information about known protein homologs and protein domains. Using the Trinotate pipeline for functional annotation of transcripts, we then detected 59,078 known transcripts in our assembly that clustered into 22,251 genes. Finally, we explored the fraction of annotated genes with TPM > 0 that are common between replicates. We retrieved a total of 18,229 genes with expression in all three replicates as well as 2680 and 448 genes expressed only in brain and heart samples, respectively (Fig. 3).

Gene Ontology analysis

**Fig. 4.**
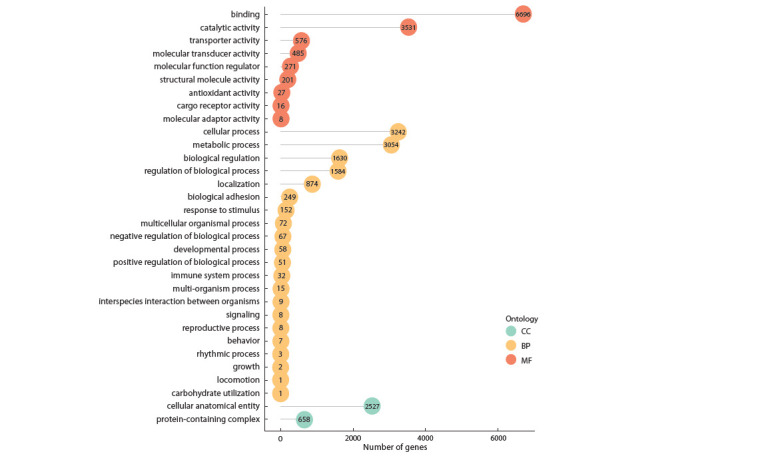
Distribution of the gene number per GO category in the transcriptome of R. amurensis. Red circles represent molecular function (MF) terms, yellow circles represent biological process (BP) terms, and light green circles show terms related to the cellular component (CC) sub-ontology.

During the annotation step, we identified a total of 11,482 genes for which corresponding GO categories were described. In particular, we found 6696 genes involved in binding (including 2988 genes associated with protein binding and 878 genes responsible for DNA binding), 3531 genes involved in catalytic activity, and 576 genes associated with transporter activity (Fig. 4).

For 10,769 annotated genes without assigned GO categories, we performed an additional analysis of their functional roles. Among them, we found several large functional gene groups, including 1969 genes associated with the RVT_1 (Reverse transcriptase) family, 1365 genes encoding zf-C2H2 (zinc finger) protein domains, and 455 genes belonging to the endonuclease/exonuclease/phosphatase family

Neurotransmitter receptor genes

A search for neurotransmitter receptors recovered a total of 47 transcripts belonging to six classes (Table 2). All detected transcripts could be unambiguously attributed to particular classes of receptors. Unexpectedly, we failed to detect any transcripts of histamine receptors. Our blastn and blastx search for these genes in the available ranid genome and transcriptome data resulted in no expressed histamine receptors in any sequenced cDNA data from any tissue. However, the genome of R. temporaria was found to contain the full gene set of histamine receptor genes. This may indicate that the histamine pathway has very limited expression in the family Ranidae.

**Table 2.**
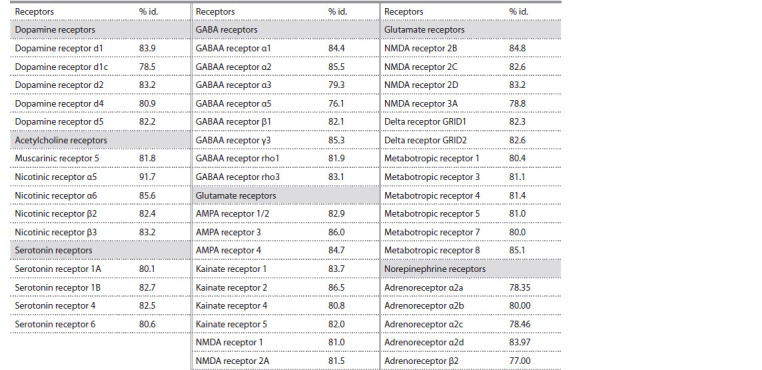
Transcripts of neurotransmitter receptors detected in R. amurensis transcriptome Note. % id. shows the percentage of identity to the respective X. tropicalis transcript along the alignable part.

Neurotransmitter receptor genes

A search for neurotransmitter receptors recovered a total of 47 transcripts belonging to six classes (Table 2). All detected transcripts could be unambiguously attributed to particular classes of receptors. Unexpectedly, we failed to detect any transcripts of histamine receptors. Our blastn and blastx search for these genes in the available ranid genome and transcriptome data resulted in no expressed histamine receptors in any sequenced cDNA data from any tissue. However, the genome of R. temporaria was found to contain the full gene set of histamine receptor genes. This may indicate that the histamine pathway has very limited expression in the family Ranidae.

The information on neurotransmitters is of special interest because they are known to be involved in hypoxia response in various organisms. G.E. Nilsson et al. (1990, 1991) found that levels of different neurotransmitters in the brain and other organs changed significantly upon exposure to hypoxia: the concentrations of GABA increased, and those of glutamate decreased in the crucian carp and the red-eared slider turtle, but not in the hypoxia-intolerant species. The authors also found that the levels of serotonin, dopamine, and norepinephrine remained unchanged, although their synthesis is oxygen-dependent. This response probably involves not just an upregulation of neurotransmitter synthesis, but the rearrangement of the whole pathway, and thus the obtained transcriptome data will be of particular use to elucidate this issue.

## Conclusion

In recent years, transcriptome analysis is increasingly used for amphibians, e. g., to study the effects of pathogens (Price et al., 2015; Xu et al., 2017), insecticides (Ma et al., 2018), or the changes occurring during metamorphosis (Birol et al., 2015; Zhao et al., 2016). Many of those studies combine data from different tissues to obtain a more or less comprehensive set of transcripts expressed in the most important organs (Yang et al., 2012; Qiao et al., 2013; Robertson, Cornman, 2014; Christenson et al., 2014). In this study, we sequenced and assembled the transcriptome of the Siberian frog R. amurensis. We also provided a quality assessment of the obtained assembly and characterized the functional roles of annotated transcripts. The available information on amphibian transcriptomes is still limited; therefore, our dataset contributes to the understanding of genome functioning and evolution of amphibians. Moreover, the majority of the previously published transcriptome assemblies for other species of the genus Rana, e. g., in I. Birol et al. (2015) and S.J. Price et al. (2015), are probably not the best option for studying the mechanisms of the hypoxia tolerance in these species. Because these studies do not focus on hypoxia and are not based on hypoxia samples, the assembled transcriptomes might miss or under-represent some transcripts specific for hypoxia. In contrast, our work creates a useful resource for studying the mechanisms of the tolerance of R. amurensis to hypoxia.

## Conflict of interest

The authors declare no conflict of interest.
